# Experiences and disease self-management in individuals living with chronic kidney disease: qualitative analysis of the National Kidney Foundation’s online community

**DOI:** 10.1186/s12882-022-02717-7

**Published:** 2022-03-04

**Authors:** Yan Du, Brittany Dennis, Valerie Ramirez, Chengdong Li, Jing Wang, Christiane L Meireles

**Affiliations:** 1 Center on Smart and Connected Health Technology, School of Nursing, UT Health San Antonio, 7703 Floyd Curl Dr, San Antonio, TX 78229 USA; 2 College of Nursing, Florida State University, Tallahassee, Florida USA; 3 School of Nursing, UT Health San Antonio, San Antonio, TX USA

**Keywords:** Chronic kidney disease (CKD), Information support, Emotional support, Online forum, Qualitative research, Self-management

## Abstract

**Background:**

Self-management of chronic kidney disease (CKD) is one of the keys in improving CKD outcomes and quality of life. There has been an increased use of online health communities to share the experiences of those living with CKD. By analyzing the CKD online forum data, this study aims to: 1) understand the experiences and challenges of individuals living with CKD, and 2) explore how online communities may help CKD patients in improving CKD self-management.

**Methods:**

Publicly available posts of peer interactions on the National Kidney Foundation’s online community for individuals affected by CKD were extracted in April 2021 using computer programming. A total of 20,436 posts were collected, of which 400 posts were analyzed using inductive thematic analysis, and saturation was reached. Two researchers coded each post independently, and discrepancies were discussed to reach consensus.

**Results:**

The analysis identified seven themes: 1) *Dynamics of CKD status,* 2) *CKD comorbidities,* 3) *Managing CKD and symptoms,* 4) *Life participation and outlook;* 5) *Navigating healthcare and clinical needs,* 6) *Medical tests and results;* and 7) *Support on the forum.* The results revealed that comorbidities were common in CKD patients and early-stage CKD was not communicated in a timely manner to patients by the health care community; living with CKD challenged both CKD and caregivers; some common challenges included but were not limited to the management of a diet for CKD and co-morbidities (especially co-morbid diabetes), CKD dynamics and symptoms, and fear of/ways to prevent progression. Individuals living with CKD primarily used the online forum to share and seek information and emotional support for managing CKD (including co-morbidities).

**Conclusions:**

Challenges of living with CKD were found not only in those with advanced kidney disease and those on dialysis, but also in those with early and middle stages. Information and emotional support from the online forum serve as a platform to empower CKD individuals with the knowledge, skills and confidence for CKD self-management. Proactive and innovative strategies with a combination of virtual and real settings to improve self-management for individuals with all-stage CKD needs to be explored and tailored.

## Background

Chronic kidney disease (CKD) is a major public health concern worldwide. The estimated prevalence of CKD is 13.4% (11.7–15.1%) globally [1]. In the United States, approximately 15% adults are estimated to have CKD in 2021 [2]. CKD is progressive and classified as stages from 1 to 5, with stage 5 being end-stage kidney disease requiring renal replacement therapy (e.g., dialysis, transplantation). CKD raises the risk of various medical complications (e.g., cognitive impairment, cardiovascular disease), and the development and progression of these medical complications increase with the severity of CKD [3, 4]. In addition, CKD is associated with poor quality of life, early death, and heavy family and health care burdens [1, 5–7]. Therefore, strategies to prevent CKD complications, slowdown/prevent progression, and improve quality of life are needed.

Self-Management of CKD is one of the keys to improving CKD outcomes and quality of life. It is the active participation in managing one’s CKD conditions, which includes but is not limited to setting goals, monitoring and assessing the condition, adhering to treatment (e.g., medication compliance, lifestyle modification), coordinating care, decision making, and resource utilization [8–10]. An in-depth understanding of the experiences, challenges, and needs of patients with CKD is essential in improving self-management of CKD. However, this may not always be conveyed in clinical settings [11]. Qualitative research is a useful approach to collect detailed evidence on patients’ priorities, goals, and needs to change and improve practice and policy [11]. The experience of individuals living with CKD has been explored using qualitative studies. However, there is always limited information in those living with early or middle stage kidney disease [12]. Additionally, oftentimes, patients’ opinions are not well captured either due to study designs, data collection approaches, or recall bias.

There has been an increased creation of social media and online health communities to support those living with certain diseases [13]. Emerging studies have reported social media and online communities as promising approaches to promote CKD care through various ways such as for data collection and providing support. For instance, Ma and colleagues collected survey data via polycystic kidney disease related Facebook groups to explore the information needs of individuals with polycystic kidney disease and their caregivers [14]. Another study also assessed the information needs of individuals with CKD, but focused on IgA nephropathy by analyzing the posts and comments of groups users of the UK IgA nephropathy Facebook group [15]. In addition, a study in UK assessed the importance of a localized online network in supporting its members dealing with kidney disease, including patients, families and caregivers [16]. The study reported that group members benefit from the online network in a variety of ways, including but not limited to information about CKD, positive mental health especially during the COVID-19 pandemic, and a localized support network to improve CKD care. However, the previous studies either focused on a specific CKD type and/or focused on a specific demographic region.

The National Kidney Foundation (NKF), a major voluntary health organization in the United States, looks to support individuals with kidney disease by offering education, support, and resources to individuals affected by CKD including patients and their caregivers [17]. To fulfill this, the NKF offers online communities for patients with CKD of any stage and caregivers alike across the world to participate in a supportive forum for individuals to share experiences, communicate and ask questions [17]. By analyzing the NKF CKD online forum data, the current study aims to 1) understand the experiences, challenges, unmet needs, goals and preferences of individuals living with CKD of all stages and their caregivers, 2) explore the approaches empowering individuals with CKD for actively engaging in self-management, and 3) assess how an online community may help CKD patients in improving CKD self-management.

## Methods

Publicly available posts of peer interactions on the NFK’s online community for individuals affected by CKD were extracted in April 2021 using computer programming. A python script was developed to scrape all publicly available posts on the forum by that time, and a total of 20, 436 posts were collected. All extracted posts were imported into an Excel (Microsoft) file for analysis. The study was determined exempted from approval by the institutional review board of the University of Texas Health Science Center at San Antonio according the HHS regulations at 45 CFR 46 and FDA regulations at 21 CFR 56 because the study would use publicly available posts (Protocol number: 20210332NHR).

Inductive thematic analysis strategy, in which no predetermined theory, structure or framework is applied, was used to analyze the data [18]. Using a convenient sampling approach, two researchers with qualitative study experience coded each post independently from the beginning of the posts listed in the excel sheet. Each researcher used an open coding analysis strategy to seek unique or similar/repeated ideas of each post, and coded them accordingly. Following their independent coding, the two researchers met to compare, contrast, and refine existing codes until they reached consensus on codes and code definitions. Frequent repetition of codes occurred for each of the researchers after analyzing around 350 forum posts. They then continued to analyze another 50 posts and determined that saturation had been reached at 400 posts. The 400 analyzed posts were posted on the NFK forum between March 3, 2021, to April 1, 2021. A senior qualitative researcher was brought for further analysis and consulted throughout the process as needed. The three researchers grouped the codes into themes, and findings were discussed and agreed upon within the research team.

In addition, two trained junior research assistants independently reviewed the same 400 posts. They independently categorized CKD stage/status into 8 categories: CKD stage 1 through 5 based on the definition of Improving Global Outcomes 2012 Clinical Practice Guideline [19], on dialysis, after transplantation, and stage unknown. They discussed and reconciled their categorization to reach agreement.

## Results

### Descriptive results

Descriptive analysis found that the 400 posts were generated from 94 unique forum users (either patients or caregivers) encompassing 50 threads. Based on the descriptions of the posts (e.g., self-reported stage, lab results), the estimated CKD stage/status of these users or users’ care recipients are shown in Table 1. Specially, 3 living with stage 1, 4 with stage 2, 20 with stage 3, 9 with stage 4, 1 with stage 5, 4 on dialysis, and 6 after transplantation. We were not able  to determine the CKD stage/status for 47 individuals. Demographic information, such as age, gender, region of residency of the forum users is not publicly available and therefore could not be identified.

**Table 1:** Estimated CKD stage/status for forum users (either CKD patients or their caregivers) of the included posts

CKD Stage/Status	Number
1	3
2	4
3	20
4	9
5	1
On dialysis	4
After transplantation	6
Stage unknown (Stage cannot be determined from posts)	47
Total	94

### Themes

The analysis of the 400 posts identified seven themes: 1) *Dynamics of CKD status,* 2) *CKD comorbidities*, 3) *Managing CKD and symptoms,* 4) *Life participation and outlook;* 5) *Navigating healthcare and clinical needs*, 6) *Medical tests and results*; and 7) *Support on the forum.* The themes and corresponding codes are show in Table 2.

**Table 2:** Themes and corresponding codes

Themes
1. Dynamics of CKD status
Current health/ disease status
Decline in health/progression of CKD
Kidney transplant
Dialysis
Improvement in overall health and CKD
2. CKD Comorbidities
Controlling comorbidities
Uncontrolled comorbidities
Underlying causes of CKD
Mistaking CKD and comorbidity symptoms
3. Managing CKD and symptoms
CKD symptoms
Medications and supplements
Diet
Exercise
Other Lifestyle management
4. Life participation and outlook
CKD and work or family
Stress
Fearful or negative feelings
Future with CKD
Optimistic/positive outlook or motivated to change health
Being unsure or resources not applying (or under support on forum for CKD benefit)
5. Medical tests and results
Lab results
Medical tests/procedures
Unexpected test results or worrying from doctor visit
6. Navigating healthcare and clinical needs
Medical history
Healthcare differences and resources
Not aware of early-stage CKD
Dissatisfied with care or not getting adequate care
Change in care
Positive remarks for doctor or healthcare
Medical specialists
COVID and CKD
7. Support on the forum
Seeking advice or support on forum
Information Support on forum
Emotional Support on forum
Consulting with or knowing what to ask doctors (other users telling others what to ask)
Slowing or preventing progression of CKD (users sharing their own or telling others on ways to slow the progression – could instead be under strategies)
Strategies for increasing CKD recommendation adherence
Caregivers

### Dynamics of CKD status

This theme focused on the dynamic range in health status and changes occurring for CKD and comorbid conditions. These included an overall improvement, decline or progression in CKD or comorbidities, such as an increase or decline in eGFR over time. It also included the dynamic thoughts and needs for procedures impacting CKD status such as dialysis and kidney transplants, including opinions on dialysis and navigating the inactive vs active transplant list. Example quotes of this theme are displayed below. To protect forum users’ privacy, we used ellipsis or “xx” to replace some statements, phrases or numbers in all cited quotes.

*“My GFR was xx last Feb, xx … My GFR is up to xx and will hopefully continue to improve.” (CKD stage 3)*

*“It's always sobering when one goes on dialysis but it does save lives. My xx (care recipient), who was very ill … , is now on dialysis and it has improved his quality of life significantly...” (Caregiver of dialysis patient)*

*“They put my xx (care recipient) on the inactive list at xx (age) so he could claim pediatric points and time accrued since he would most likely be an adult when the kidney was needed. I thought it was a good … for him. Can be upgraded to active at anytime and reviewed at least once xx year.” (Caregiver of CKD stage 4)*

### CKD comorbidities

This theme focused on the interplay and connections between CKD and comorbidities or the impacts that one can have on the other. Users discussed trying to control comorbidities through methods such as medication, to ultimately have a positive impact on CKD. Users also discussed uncontrolled comorbidities or those chosen not to be managed to focus attention on CKD. The connections between CKD and comorbidities were also seen in relation to underlying causes for CKD, such as hypertension and diabetes, as well as mistaking CKD and comorbidity symptoms for that of the other condition which could delay treatment of the proper cause. Example quotes of this theme are displayed below:

*“My HBP [high blood pressure] is also controlled by meds and the diabetes is controlled with my diet and exercise.” (CKD stage3)*

*“I'm a caregiver to my xx (care recipient), a Diabetic Type 2, who is now on dialysis. Unfortunately, diabetes destroys the small blood vessels inside your kidneys. In turn, that erosive force can also create heart disease, … blood pressure, and more.” (Caregiver of dialysis patient)*

*“My BP, CKD, diabetes, Osteoporosis, IBS, detached upper arm muscles, high cholesterol and brain fade from xx are all untreated. ...” (CKD stage unknown)*

As these posts were originally posted on the forum during the COVID pandemic, some posts discussed COVID related matters, including but not limited to getting the vaccine while having CKD and comorbidities.

*“Ckd and covid vaccine!?? Anybody here got the covid shots … is it safe for xx and ckd patient? what do you feel after the shots? Thnk you !” (CKD stage unknown)*

### Managing CKD and symptoms

This theme focused on CKD symptoms, as well as the strategies and challenges of their management. CKD symptoms shared through the forum were dynamic and varied between individuals. Discussed symptoms included but were not limited to fatigue, edema, stress, anxiety, exhaustion, itchy skin and general discomfort associated with CKD. Users often shared their own ways of how to manage symptoms that others were experiencing, such as advice on managing itchy skin by using a moisturizer or taking a bath before bed.

*“I'm extremely tired and have no energy, xx cloudy urine, xx pain, xx pain, dizziness, brain fog, weakness, just xx so exhausted.” (CKD stage unknown)*

*“I am stage 3 B and feel so tired that I have to take xx in the morning to get going. … the fatigue is debilitating. I just sit the rest of the day. Everyday I try to use a xx bike. I rarely can do more that a few minutes at a time. Does this sound like your experiences,” (CKD stage 3)*

*“Sorry to hear your xx is having this problem [skin itchiness interfering with sleep]. The best thing I found was to have a nice warm bath before bedtime. While in the bath I would …. Then I would …. It used to help me sleep better.” (CKD stage unknown)*

Management of CKD incorporated a range of challenges, approaches and techniques individuals used or recommended to others such as diet, exercise, medications, supplements, and lifestyle; amongst other strategies. Diet was the most frequently discussed, which also had its own challenges and strategies. Challenges included diet recommendations for CKD and diet recommendations for other comorbid conditions conflicting, while strategies for managing CKD with diet ranged from a vegetarian diet, no added salt diet, to using whole grains over refined carbohydrates, and what the optimal level of protein consumption is.

*“Taking certain meds that work at cross purposes is a difficult situation. For me it's xx. NSAIDs are not recommended for xx with CKD.” (CKD stage 2)*

*“I'm in stage 3 [CKD]... The things that they recommend on the kidney disease diet such as xx and xx are an absolute NO on the diabetic diet. Everything contradicts each other…” (CKD stage 3)*

*“My meal plan was designed specifically for me based on my diabetes, kidney disease, and xx. Low sodium foods and I use herbs and spices for flavor instead of salt. … No sweets and nothing that turns into bad carbs when digested. … Portion control is critical and by far the largest portion on my plate is the veggies. If you can, obtain a referral to meet with a renal dietitian and bring your …, and design something for you.” (CKD stage 3)*

### Life participation and outlook

As there are many challenges and aspects to managing CKD, this theme focused on how an individual’s life or outlook on life was affected by living with CKD, both positively and negatively. Life participation topics included CKD impacts on work, stress, emotions and family. Examples included needing to take time off from work due to not feeling well from CKD symptoms or side effects and dealing with stress directly from CKD management or from external sources.

*“In the last xx months in my opinion my health has decreased considerably such as not having any energy and not appetite which has caused me to take xx time off work.” (CKD stage 4)*

“…*I'm often too tired to do anything more and my xx hr/wk job. I argue with xx about my lethargy and lack of motivation for activity. I doubt I'm alone in this. Thank you for being here.” (CKD stage 3)*

Life outlook included past, present or imagined future feelings, such as negative or fearful emotions around diagnosis or living with CKD, outlook on the future with CKD, and being unsure of what one should do for their own CKD care. Other topics included having an optimistic outlook on life with CKD, being motivated to change one’s own health or feeling as though health is within one’s own control.

*“It's just that I'm worried everytime I see xx in my urine, I get all these negative thoughts about my kidney not lasting long.” (CKD stage unknown)*

*“We can each maximize our own situations. Regardless of treatment approach, doing this will improve our quality of life.” (CKD stage unknown)*

*“I learned that GFR decreases at a rate of xx every year after the age of xx even for normal healthy people. I’m xx years old now and my GFR is xx, although everything is stable. Do I get to live to xx? Do I get to see my grandchild? My daughter is only xx....Will I be on dialysis in my xxs?... All these questions just keep lingering in my minds...” (CKD stage 3)*

### Navigating healthcare and clinical needs

This theme included discussions, statements, and navigation of medical and clinical needs an individual with CKD experiences. These clinical needs included any type of healthcare professional or specialist as well as generalized to specialized needs, which could be impacted based on an individual’s specific comorbidities, challenges with management, life participation or outlook, and needs during the COVID pandemic. Both positive and negative remarks in relation to clinical needs as well as users discussing questions or giving advice based on their own experiences for medical and clinical needs were found within this theme. Examples included healthcare differences such as how care differs across country borders, an individual being dissatisfied with their care or not getting adequate care, being pleased with healthcare or doctor and using medical specialists such as a dietitian or nephrologist to guide them within their CKD journey.

*“I'm in the xx (country) so things are a bit different over here - the xx (country) seem much more proactive about CVD, CKD etc. I wish I could get more answers - I emailed the cardio but he has just ignored it… I've never been so frightened in my life - I've tried reading everything ... Thanks for your help - even just hearing from someone helps to be honest!” (CKD stage 3)*

*“Nephrologist’s in many states in the xx (country) refuse to see you until stage 4. I got in because I played the hey i only have one kidney xx, and I earned it. She was xx, rude, and seemed to think I was overreacting to a gfr gone from xx to xx, repeatedly, ...” (CKD stage 4)*

*“With COVID I’ve had several telemedicine (phone) appointments too. I am able to show my physicians not only my face but also my hands, xx, lower legs, etc, during these appointments. It’s not as good as a physical exam where my doctors can actually feel for edema, but it has worked. They also have me send them a table with my xx readings.” (CKD stage unknown)*

Another frequently discussed topic was individuals not being aware of early-stage CKD, in which users described not having been told about being in early-stage CKD until it had progressed farther, reducing the early ability for progression prevention. They also discussed that some health specialists may not adopt a proactive approach for

*“Found out i had CKD stage 3b from my labs and office visit report. I believe this has happened to many people on this forum. Not sure why primary doctors don’t address this condition earlier so we can start trying to delay the progression.” (CKD stage 3)*

*“I'm somewhat angry because I've been diagnosed as CKD Stage 3a and my primary care doctor very nonchalantly said "oh you're Stage 3 kidney disease. You've been having some high creatinine and low GFR a couple times in the past couple years." For goodness xx! I went from not knowing anything about CKD to Stage 3a. Cripes. What happened to Stage I and 2?” (CKD stage 3)*

*“… I'm a mess. I was diagnosed 2 years ago with diabetes xx and that and the fact that I have high blood pressure (although now very controlled) lead me to kidney disease. I'm in stage 3. The thing is, I didn't even know that I was in stage 1 or 2. A million specialists and nobody said a word. I'm terrified and confused” (CKD stage 3)*

### Medical tests and results

This theme focused on the medical and lab tests which individuals with CKD frequently require to manage and monitor CKD. This included lab results, in which users would often post their recent lab results to give others indication to their health and open the floor to advise on how to better manage. It also included medical tests and procedures such as biopsies, as well as individuals receiving unexpected test results such as seeing renal failure indicators or being worried about health or CKD after a doctor’s office visit or while waiting on test results.

*“Has anyone else had this? When I was diagnosed with CKD I had a kidney biopsy and they advised they would take 2-3 samples. Unfortunately the samples were all fragmented and they ended up taking xx samples… I am concerned my kidneys are totally destroyed as they couldn’t get one clear sample.” (CKD stage 3)*

*“I have received a lab work but I do have question on the range. So my albumin was xx. So even though it is in normal range but it went up from xx (previous lab) to xx (current lab). Is that a sign of concern that it went up and hovering around closer to 5 and eventually be out of range?” (CKD stage unknown)*

### Support on the forum

This theme encompasses the many types of support between peers communicating within the online forum. Support was often bidirectional as users both gave advice and went to the forum to seek advice from others, in addition to sharing stories, experiences and tips for living with CKD. Support came in many forms and included both individuals with CKD and caregivers for someone with CKD alike. Information support included providing specific information within a post, such as specific books, websites or specialists to look see, while emotional support included supporting or empathizing with others’ emotional journey, frustrations, or successes. Examples included users educating others in knowing what to ask doctors during their next appointment, expressing plans or recent consultations with doctors, as well as strategies for slowing progression of CKD, and strategies for increasing CKD recommendation adherence. Example quotes from this sub-theme are displayed below:

*“I need help with finding a cookbook or website with recipes for unimaginative cooks. Also, any ideas on what are good replacements: say, recipe calls for beans, what would be a good substitution.” (CKD stage 4)*

*“When you meet with a nephrologist ask for a referral to meet with a Renal Dietitian and bring as many hard copies of your labs … and together you and the RD can develop a kidney-friendly meal plan based on your …. Once you know where you stand, come back as often as you need for advice and support...” (CKD stage unknown)*

*“No thanks ever needed; my pleasure. You have come to a community of wonderful, compassionate and knowledgeable people who are happy to share and support.” (CKD stage unknown)*

## Discussion

By qualitatively analyzing the posts of the CKD online forum, our study found that the experiences and challenges of individuals living with CKD depended on many factors (e.g., CKD status and comorbidities, symptoms, clinical and health care needs), all of which are impacted by situation specific needs and goals throughout an individual’s journey. The online forum provides a convenient platform for CKD patients and their caregivers to share experiences and challenges of living with CKD and seek information and emotional support on demand, and in a flexible timeframe. Individuals expressed, relayed, and gained knowledge through supporting each other on the forum, looking to ultimately have a positive impact on and improvement in CKD management. We acknowledge that the CKD online forum plays a significant role in educating, engaging, and empowering CKD patients and their caregivers to manage CKD and ease anxiety and stress. Meanwhile, more proactive strategies are needed in the real setting for early CKD detection, communication, and management advice and consultation. Although there are some general, common needs CKD patients at different stages share, there are also many unique needs, symptoms, and personal characteristics. Therefore, individualized and tailored approaches to improve self-management should be recommended.

Based on the discussions on the forum, individuals living with CKD often have one or more comorbidities, which may be risk factors for CKD progression and increase the treatment and self-management burdens on CKD patients. This phenomenon has been reported by prior quantitative studies [4, 20, 21]. Diabetes and high blood pressure are the most common comorbidities of CKD, and the Kidney Disease: Improving Global Outcomes (KDIGO) practice guidelines have been issued periodically to improve the management of the comorbid conditions [22, 23]. Despite the available guidelines, forum users frequently discussed the challenges of managing CKD comorbid with diabetes and/or high blood pressure regarding medication and/or lifestyle management. Therefore, understanding each CKD patient’s health conditions and their challenges of living with CKD and comorbidities is essential to develop tailored and effective management strategies.

Diet management was one of the most challenging self-management tasks revealed by this study. It has been widely evidenced that diet regimen in CKD patients vary by an individual’s CKD stage or remaining kidney function. For example, depending on one’s lab results, some food items high in potassium or phosphorus might be limited. On the forum, the meaning of lab results was also frequently discussed, which were sometimes attributed to diet choices. This may highlight that managing CKD diet may require a higher level of health literacy, reflected in the forum by high frequency of lab result discussions. Meanwhile, peers in the forum also suggested to bring lab results to a dietitian for an individualized diet plan, especially for those living with comorbidities. Living with CKD and comorbidities further complicates the diet regimen. For example, forum users discussed the conflicting diet regimen of diabetes and CKD. Due to the increasing needs of managing comorbid diabetes and CKD, in 2020, the KDIGO issued the first version of practice guidelines of managing diabetes in CKD patients involving both pharmacological and lifestyle management. In the guideline, the complexity of diet management is acknowledged. The guideline points out that individualized and tailored diet regimen should be implemented. However, how to individualize and tailor diet regimen is not conveyed, and still needs to be explored further.

Their symptoms and lab results (e.g., renal function) may independently or jointly influence patients’ life participation and outlook. Individuals with CKD often experience a variety of symptoms, especially as CKD progresses. As reported previously, fatigue, anxiety and depression symptoms are particularly prevalent in those with end-stage renal disease and/or on dialysis [24, 25]. The various symptoms may interact and influence on each other, and impact one’s life participation and outlook for future. For example, fatigue leads to sedentary lifestyle and social participation, which may cause depression and further renal function decline. However, as relayed by forum users, whether the symptoms are caused by CKD and/or its comorbidities is often hard to be determined by patients themselves, as well as by health professionals [26]. Therefore, clustering CKD symptoms and correlating symptoms with various health conditions may help improve CKD symptom management, and improve patients’ health outcomes and quality of life. In addition, the current study found that CKD patients of moderate stages (e.g., stage 3 and 4) also suffer from various CKD symptoms, which calls the scientific and clinical’s attentions to promote interventions for symptom management in earlier CKD stages.

Notably, our study revealed that CKD patients experience a wide array of problems, but felt unheard, ignored, and misunderstood by their healthcare professionals. Forum users also frequently complained that they were not informed about CKD when it was in early stages. According to the CDC, as much as 9 in 10 adults in the US with CKD don’t know they have it, and around 2 in 5 adults with severe CKD are not aware that they have CKD [27]. This is a serious problem since if not managed, CKD may progress into later, more severe stages, and the subsequent cost and burden to both the individual and the healthcare system would be much higher compared to taking action in earlier stages [28, 29]. Actions for early CKD detection and prevention should be taken not only by the primary health providers, but also by public health professionals, to encourage earlier CKD detection and communication about the diagnosis with patients in a timely manner. Furthermore, early action and education can help raise awareness of CKD severity, which is important as patients can be symptom driven when managing health conditions, and consequently those newly diagnosed with early-stage CKD may not care about their conditions due to seeing minimal or no symptoms at the time. Shared decision making between health care providers and CKD patients is the cornerstone of eliciting self-management. Patients share information about their experiences of illness, values, and preferences while clinicians complement with information and recommendations with each individual patient in mind [30]. CKD will continue to be a major public health concern, as the aging population living with diabetes and hypertension, the two major risk factors of CKD, are rapidly increasing [1]. Proactive strategies to detect and communicate early-stage CKD, improve decision making to initiate CKD self-management and empowering patients to manage their CKD are urgently needed. Meanwhile, whilst supportive of early action and education, having resources available when patients develop the need remains important. Social media, including online forum, which is usually available 24 h a day/7 days a week, is helpful as a supplement of resources in the real-world settings in supporting patients with resources and information when individuals have a need.

Similar to other online health communities, our study found that CKD forums have become an important community to empower CKD patients for CKD self-management through various support. Our findings suggest that despite from various regions around the world, the forum commutations may empower forum users in improving the fundamental self-manage skills such as problem-solving, decision making, and communicating with healthcare professionals. The forum also empowers forum users with knowledge and efficacy through informational and emotional support to improve the skills and confidence of managing their CKD. These findings suggest that forum users are actively seeking ways to improve patient activation in CKD self-management. The widespread and easy access of social media may enable both active and less active forum members expose to their needed information. To be noted, access to internet may vary by demographics. For example, higher education may be associated with higher access to internet and consequently more resources to improve their health conditions. This may lead to further health disparities. Future interventions to improve CKD self-management may consider a combination of in person and online strategies tailored to individual factors such as CKD status, individual and clinical needs, and demographics.

## Limitations

There are several limitations of the study. First, the forum users were from across the world. The results might not be applicable to a specific country or region. However, this is also a strength of this study of assessing the experiences and challenges of CKD patients from a very diverse background globally. Second, the online forum is in English, so the transferability of our findings to non-English speaking CKD patients is uncertain. Third, all data was collected via an online forum, and the findings may not be generalizable to those who have limited access to internet and/or computers. Meanwhile, communities, including online communities, are largely influenced by active members [31], who may contribute often to the discussions, and therefore creating a potential ingroup bias in the online community. However, the nature of the data collection in a natural (non-research) setting of the study is a strength as well. Forth, the size of datasets is too big to be all analyzed manually. We may have missed information from the forum by only conveniently selected a small number of posts for analysis. Lastly, since it is not required when registering for an online forum account and we only accessed to publicly available data, we didn’t have data such as age, gender, or region of residence of forum users. Therefore, we were unable to relay our findings for a more specific group. Making some data information required when registering for an account to post a topic or comments may further improve and facilitate future research using forum resources. With this, guidelines and relevant policies to use such information needs to be developed to ensure forum users’ right and privacy are well protected.

## Conclusion

Despite some limitations, this study is one of the first studies assessing peer interactions of an online community with forum users across the world who are affected by CKD, regardless of CKD stages. By analyzing the user interactions on the CKD online forum, we found that not only those with end-stage kidney disease, those with earlier CKD also struggle with various challenges of living with CKD and comorbidities. The online CKD community provides a great platform for CKD patients to improve patient activation for CKD self-management through sharing information, exchanging management skills and health care coordination, and encouraging and comforting each other. Proactive intervention programs to improve CKD self-management with a combination of in person and online strategies tailored to the characteristics of each CKD patient are needed.

## Data Availability

The datasets generated and/or analyzed during the current study are available in the National Kidney Foundation’s online community repository, https://www.kidney.org/online-communities

## Abbreviations

CKD: Chronic kidney disease
HHS: Department of Health and Human Services
NKF: National Kidney Foundation
